# Perioperative effects of different hypotensive anesthesia techniques in orthognathic surgery

**DOI:** 10.4317/medoral.26662

**Published:** 2024-06-22

**Authors:** İslam Kazımlı, Canay Yılmaz Asan, Dilek Günay Canpolat, Ahmet Emin Demirbaş

**Affiliations:** 1 Resident, DDS. Department of Oral and Maxillofacial Surgery, Faculty of Dentistry, Erciyes University, Kayseri, Turkiye; 2 Associate Professor, DDS. Department of Oral and Maxillofacial Surgery, Faculty of Dentistry, Erciyes University, Kayseri, Turkiye; 3 Professor, MD. Department of Oral and Maxillofacial Surgery, Faculty of Dentistry, Erciyes University, Kayseri, Turkiye; 4 Associate Professor, DDS. Department of Oral and Maxillofacial Surgery, Faculty of Dentistry, Erciyes University, Kayseri, Turkiye

## Abstract

**Background:**

This study aimed to investigate the effectiveness of combining sevoflurane with remifentanil, esmolol, or nitroglycerin for hypotensive anesthesia and determine the suiTable hypotensive anesthesia method for orthognathic surgery.

**Material and Methods:**

This retrospective study included 60 patients who underwent orthognathic surgery for developmental malocclusion. They were divided into three groups based on the hypotensive agent preferences: Group 1 (n = 20), sevoflurane and remifentanil; Group 2 (n = 20), sevoflurane and esmolol; Group 3 (n = 20), sevoflurane and nitroglycerin. Bleeding volume, heart rate, systolic, diastolic, and mean arterial blood pressure were recorded at certain times during the perioperative period, including at stages with increased stress levels in the body, such as incision and osteotomy. The patients’ blood pressure, analgesic consumption and pain level were recorded in the postoperative period.

**Results:**

Bleeding volume, surgery satisfaction related to bleeding, and total operation time did not differ significantly between groups. Intraoperatively, heart rates were significantly higher in Group 3 than in Groups 1 and 2 (*p* = 0.001). However, hemodynamic stability was similar in Groups 1 and 2. Postoperatively, analgesic consumption, pain levels, and blood pressure dynamics did not differ significantly between groups (*p* > 0.05).

**Conclusions:**

Based on this study’s results, it was concluded that infusing remifentanil, esmolol, or nitroglycerin with sevoflurane during orthognathic surgery successfully achieved the targeted hypotensive anesthesia and can be considered alternative methods. The decision on which method to use should consider the patient’s overall health status and additional medical conditions.

** Key words:**Orthognathic surgery controlled hypotensive anesthesia, remifentanil, esmolol, nitroglycerin, sevoflurane, surgeon satisfaction, bleeding.

## Introduction

Orthognathic surgeries are used to correct diverse dentofacial deformities and have some common complications, including intra-operative and postoperative bleeding, pain, and postoperative edema (1). The rate of orthognathic surgery related complication is 9.7% (2). The complex vascular supply in the facial region makes surgical procedures challenging.

Controlled hypotensive anesthesia is a method used to reduce blood loss and minimize bleeding-related morbidity (3). The patient’s mean arterial pressure (MAP) is reduced by 30% during hypotensive anesthesia, causing systolic blood pressure (SBP) to decrease to 80-90 mmHg and MAP to 50-65 mmHg (4).

Controlled hypotension was first suggested by Cushing in 1917 (5). Various methods have subsequently been used to induce hypotension during orthopedic and neurosurgical procedures. In oral and maxillofacial surgery, hypotensive anesthesia which was first reported by Schaberg *et al* (4). In addition to orthognathic surgery, hypotensive anesthesia is preferred in many surgeries, including middle ear surgery (6), functional endoscopic sinus surgery (7), and hip arthroplasty (8). The most commonly used anesthesia agents are magnesium sulfate, nitroglycerin, vasodilators (sodium nitroprusside), beta-adrenergic antagonists, and potent inhalation anesthetics (5). Since the choice of anesthetic agent depends on the anesthesiologist, there is no standardized protocol.

This study aimed to investigate the relative effectiveness of hypotensive anesthesia methods combining sevoflurane with remifentanil, esmolol, or nitroglycerin infusion and determine the suiTable hypotensive anesthesia method for orthognathic surgery.

## Material and Methods

- Study design

This retrospective study was approved by the Erciyes University Clinical Research Ethics Committee (No: 2022/40, dated January 5, 2022). It included 60 patients who underwent orthognathic surgery for developmental malocclusion in the Department of Oral and Maxillofacial Surgery of the Faculty of Dentistry at Erciyes University between May 17, 2022, and November 1, 2022.

The inclusion criteria were patients aged ≥18 years with American Society of Anesthesiologists (ASA) I-II Class II-III malocclusions undergoing elective double jaw surgery. The exclusion criteria were a history of trauma in the craniofacial region, cleft lip and palate, congenital syndromes, craniofacial anomalies, and incomplete records.

- Study groups

The patients were randomly divided into three groups based on the agents used during their anesthesia: Group 1 (n = 20), sevoflurane and remifentanil; Group 2 (n = 20), sevoflurane and esmolol; Group 3 (n = 20), sevoflurane and nitroglycerin. The patients’ anesthesia records during the surgery and observation forms by the nursing staff and doctors in the ward were examined.

- Variables

Demographic and operation-related parameters possibly associated with hypotensive anesthesia were recorded. The demographic parameters were sex, age, weight, and height. The operation- related variables were the total amount of bleeding, total fluid balance, anesthesia duration, surgery duration, and surgeon satisfaction (measured on a Likert scale: 1 = very unsatisfied, 2 = not satisfied, 3 = neutral, 4 = satisfied, 5 = very satisfied). Hemodynamic variables such as systolic blood pressure (SBP), diastolic blood pressure (DBP), and mean arterial pressure (MAP) were recorded at the following times during the preoperative, perioperative, and 24- hour postoperative periods (T0-T20): T0, baseline before anesthesia; T1, immediately after endotracheal intubation; T2, five minutes after intubation; T3, before Le Fort I osteotomy; T4, after osteotomy of the right pterygoid plate in Le Fort I; T5, after osteotomy of the left pterygoid plate in Le Fort I; T6, after Le Fort I down fracture; T7, during Le Fort I suturing; T8, before bilateral sagittal split osteotomy (BSSO); T9, after the right split in the BSSO; T10, after the left split in the BSSO; T11, during BSSO suturing; T12, during extubation; T13, five minutes after extubation; T14, 15 minutes after admission to the recovery room; T15, 30 minutes after admission to the recovery room; T16, one hour postoperative; T17, three hours postoperative; T18, six hours postoperative; T19, 12 hours postoperative; T20, 24 hours postoperative.

Patients’ pain levels (using the visual analog scale [VAS]) and analgesic consumption were also recorded at 30 minutes and 1, 12, and 24 hours postoperative.

- Anesthesia management

The same anesthesia and surgical teams performed all surgeries. Anesthesia was induced in all groups intravenously using 2.0 mg/kg of propofol (Propofol®; Fresenius Kabi, Germany) and 0.6 mg/kg of rocuronium (Esmeron®; GlaxoSmithKline, UK). The patients were intubated using a nasotracheal approach. When necessary, 0.1 mg/kg of rocuronium was given for muscle relaxation during the operation.

Hypotensive anesthesia was used to facilitate the improvement of the surgical area and reduce bleeding. Anesthesia was maintained using 1.5% sevoflurane (Sevorane®; Abbott, USA) and a loading dose administered with remifentanil (1 mcg/kg), esmolol (500 mcg/kg), and nitroglycerin (5 mcg/kg), 50% nitrous oxide in oxygen, 50% oxygen for air (tidal volume = 6- 8 mL/kg, frequency = 10/min). Maintaining infusions of 0.5-20 mcg/kg/min for remifentanil, 100-200 mcg/kg/min for esmolol, and 0.5-2 mcg/kg/min for nitroglycerin. Attention was paid to ensuring that the patient’s average blood pressure did not drop below 60 mmHg, achieving a hypotensive state.

- Surgical procedures

All patients received bilateral buccal, inferior alveolar, and lingual nerve block anesthesia in the mandible and bilateral buccal infiltrative anesthesia in the maxilla with 2% articaine (80 mg; Ultracain 2% Ampoule; Sanofi-Aventis, Istanbul, Turkey) in addition to 1/200,000 epinephrine. Le Fort I osteotomy was performed using the Bell method (9), and BSSO was performed using the Hunsuck modification (10). A horizontal incision was made in the vestibule sulcus with a cautery between premolars in the maxilla. The mucoperiosteal flap was removed to expose the lateral walls of the maxilla, pyriform aperture, and zygomatic buttress bilaterally to the lower level of the infraorbital foramen. The nasal mucosa was elevated. A Le Fort I osteotomy cut was made using Piezo surgical saws, the pterygomaxillary junction, nasal septum, and lateral nasal wall were osteotomized, and the maxilla was downfractured using a spreader and hook. The maxilla was brought to its new planned position using the intermediate splint prepared before surgery. The maxilla was fixed in this new position using titanium miniplates and screws in the apertura piriformis and zygomatic buttress zones. A horizontal incision was made with cautery bilaterally along the oblique ridge in the mandible. The mucoperiosteal flap was raised on both sides, exposing the medial surface of the ramus until the lingula, lateral surfaces of the corpus, and angulus. Bilaterally, the medial ramus was cut horizontally just over the lingula Lindeman burs, with the oblique cut through the oblique ridge and the vertical cut in line with the medial of the second molar. The osteotomy lines were just defined by tapping with an osteotome, and then the splitting was completed using a spreader and lower border separator. The mandible was moved to its new position using the final splint prepared before surgery and fixed with a miniplate and titanium screws.

After the surgical procedure was completed, 1 mg of neostigmine (Neostigmin® Ampoule 0.5 mg/mL; Adeka, Samsun, Turkey) and 0.5 mg of atropine (Atropine Sulphate® Ampoule 0.5 mg/mL; Galen, Istanbul, Turkey) were administered to reverse the muscle relaxants. The surgery duration, total intraoperative bleeding, and any complications were documented. Next, the patient was extubated and transferred to the post-anesthesia care unit. In the first 24 hours after surgery, 73.8 mg of dexketoprofen trometamol (equivalent to 50 mg of dexketoprofen; Arveles 50 mg/2 mL; UFSA Pharmaceuticals, Turkey) was administered intravenously as needed for pain control. In cases where dexketoprofen proved insufficient and additional anesthesia was required, 1000 mg of paracetamol (Paracetamol 10 mg/mL [100 mL vial]; Bristol-Myers Squibb Pharmaceuticals, Turkey) and 100 mg of tramadol (Tramadol 100 mg Ampule; Abdi İbrahim Pharmaceuticals, Turkey) were administered intravenously as the second choice. Postoperatively, 1 g of cefazolin (Cezol; Deva Holding, Turkey) was administered intravenously twice daily to prevent postoperative infection, and 4 mg of dexamethasone was administered to control edema.

- Data analysis

The data were analyzed using SPSS software (version 23; IBM Corp., Armonk, NY, USA). The normality of the data distribution was assessed using the Shapiro-Wilk test. Categorical variables were compared across groups using the Chi-square test. Non-normally distributed variables were compared across groups using the Kruskal-Wallis test and between two groups using Dunn’s test. Normally distributed variables were compared across groups using one-way analysis of variance (ANOVA) and between two groups using Duncan’s test or Tukey’s honestly significant difference test. Quantitative variables are presented as the mean ± standard deviation or median (minimum-maximum), while categorical variables are presented as frequencies (percentages). A *p* < 0.05 was considered statistically significant.

## Results

- Demographic results

This study included 60 patients divided into three groups: Group 1 comprised eight males (40%) males and 12 females (60%), Group 2 comprised 10 males (50%) and 10 females (50%), and Group 3 comprised five males (25%) males and 15 females (75%). The median weight and height were 66.50 kg and 168.00 cm in Group 1, 67.50 kg and 170.00 cm in Group 2, and 56.50 kg and 165.00 cm in Group 3, respectively. The sex, weight, and height distributions did not differ significantly between groups (*p* > 0.05). However, the age distribution differed significantly among groups (*p* = 0.021), with a median of 22.50 years in Group 1, 23.50 years in Group 2, and 19.00 years in Group 3. Therefore, this disparity arises from the difference between Groups 2 and 3.

- Operation related results

The median total blood loss was 400.00 mL in Group 1, 450.00 mL in Group 2, and 400.00 mL in Group 3 (*p* = 0.354). The mean intraoperative fluid replacement was 1912.50 mL in Group 1, 2110.00 mL in Group 2, and 1867.50 mL in Group 3 (*p* = 0.189). Anesthesia duration did not differ significantly between Groups 1 (median = 240.00 min), 2 (median = 245.00 min), and 3 (median = 220.00 min; *p* = 0.412). The mean surgical duration was 219.00 min in Group 1, 223.25 min in Group 2, and 213.75 min in Group 3 (*p* = 0.820). Perioperative total bleeding volume, amount of intraoperative fluid replacement, anesthesia duration, and surgical duration also did not differ significantly between groups (*p* > 0.05; Table 1).

- Postoperative pain level

**Figure 1:**
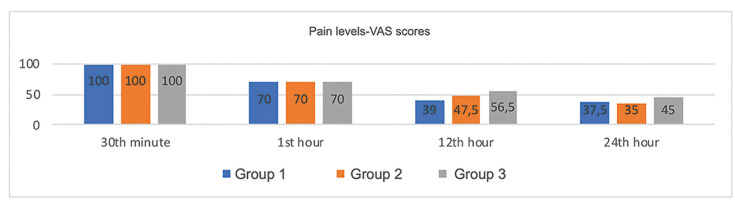
Postoperative pain levels among groups. Kruskall-Wallis H test: 30 minutes (*p* = 0.987), 1 hour (*p* = 0.917), and 24 hours (*p* = 0.563) postoperative; ANOVA: 12 hours (*p* = 0.154).

The mean VAS scores 30 minutes and 1, 12, and 24 hours postoperative were 100.0, 70.0, 39.0, and 37.5 in Group 1; 100.0, 70.0, 47.5, and 35.0 in Group 2; and 100.0, 70.0, 56.5, and 45.0 in Group 3, respectively. Therefore, VAS-based postoperative pain levels did not differ significantly between groups (*p* > 0.05; Fig. 1).

-Postoperative dexketoprofen consumption

**Figure 2:**
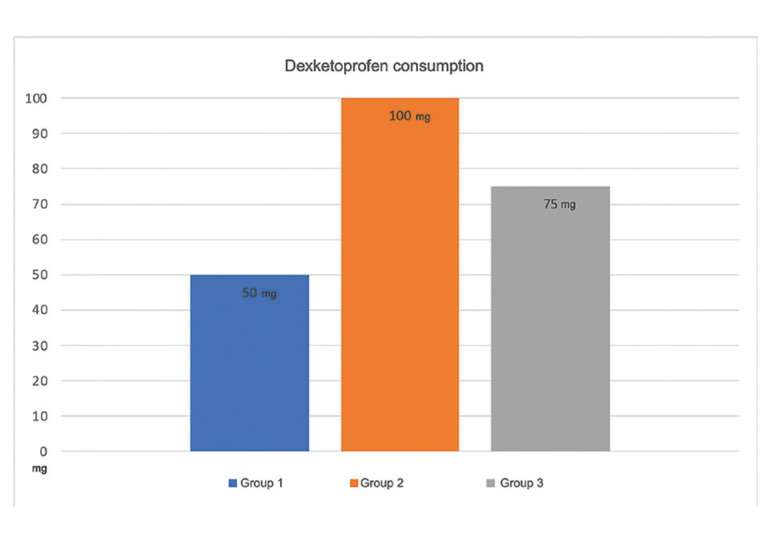
Postoperative dexketoprofen consumption. Kruskall-Wallis H test: *p* = 0.542.

The median dexketoprofen dose was 50.00 mg in Group 1, 100.00 mg in Group 2, and 75.00 mg in Group 3. Despite the higher consumption of dexketoprofen in Group 3, there was no statistically significant difference between groups (*p* = 0.542; Fig. 2).

- Perioperative and postoperative heart rate dynamics

**Figure 3:**
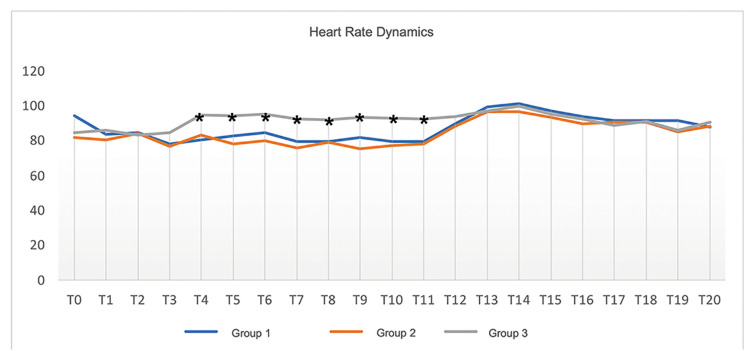
Perioperative and postoperative heart rate dynamics. Key: *, *p* < 0.05.

Intraoperative heart rates were higher in Group 3 than in Groups 1 and 2 (Table 2; Fig. 3), with a mean rate of 94.70 ± 13.73 at T4, 94.20 ± 14.70 at T5, 95.05 ± 13.52 at T6, 92.45 ± 14.57 at T7, 92.00 ± 14.24 at T8, 93.15 ± 10.92 at T9, 93.00 ± 10.07 at T10, and 92.20 ± 11.78 at T11.

- Perioperative and postoperative hemodynamic results

**Figure 4:**
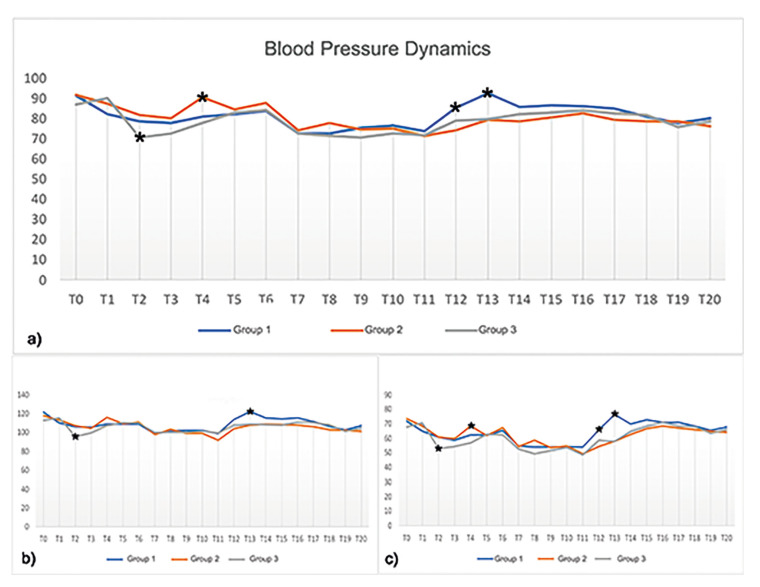
Perioperative and postoperative MAP (a), SBP (b), and DBP (c) dynamics. Key: *, *p* <0.05.

Blood pressure dynamics followed the same pattern in all three groups (Table 3; Fig. 4). At T2, the median MAP was higher in Group 3 (69.00 mmHg) than in Groups 1 (81.50 mmHg) and 2 (83.00 mmHg), indicating greater hypotension. In contrast, the median MAP was higher in Group 2 (90.65 mmHg) than in Groups 1 and 3 at T4. Furthermore, MAP reached normotensive levels at T12 and T13 more rapidly in Group 1 (85.30 and 92.50 min, respectively) than in Groups 2 (74.20 and 79.25 min, respectively) and 3 (79.05 and 79.90 min, respectively).

- Surgeon satisfaction

The median surgeon satisfaction score was 4.00 for Group 1, 4.00 for Group 2, and 4.00 for Group 3. Therefore, surgery satisfaction did not differ significantly among groups (*p* > 0.05).

## Discussion

Orthognathic surgeries performed to correct various dentofacial deformities cause significant blood loss, which cannot be controlled primarily by identifying and ligating vessels (11). Controlled hypotensive anesthesia is a technique used to reduce blood loss and minimize potential intraoperative and postoperative complications in these surgeries.

A systematic review by Choi *et al*. found that hypotensive anesthesia effectively reduced blood loss and improved surgical field view during surgeries such as orthognathic surgeries, and serious outcomes due to organ hypoperfusion were rare with this technique (12).

During orthognathic surgery, controlled hypotensive anesthesia can be achieved with inhalation anesthetics, intravenous drugs, or their combination (13). The ideal hypotensive agent should be safe and easy to administer (14). The most commonly used agents are magnesium sulfate, nitroglycerin, vasodilators (e.g., sodium nitroprusside), beta-adrenergic antagonists, and potent inhalation anesthetics (5). While no consensus exists on which agent to use, many studies have examined and compared the advantages and disadvantages of these agents.

While studies have investigated the effects of hypotensive agents on blood loss, surgical field quality, and hemodynamics in various surgeries, including orthopedic, nasal, and middle ear surgeries, our study may be the first to compare the effects of three different hypotensive agents in orthognathic surgery.

Ayla *et al*. compared the effectiveness of nitroglycerin and esmolol in creating controlled hypotension in nasal surgery. They found that esmolol provided a good surgical field of view in controlled hypotension and induced similar hemodynamic changes to nitroglycerin (14).

Srivastava *et al*. also compared nitroglycerin and esmolol in establishing controlled hypotension in functional endoscopic sinus surgery. They discovered that both esmolol and NTG are viable and safe medications for this purpose. Nonetheless, esmolol demonstrated superiority by ensuring optimal surgical conditions with only a slight decrease in blood pressure. Additionally, it facilitated reduced intraoperative bleeding and mitigated tachycardia throughout the surgery, offering supplementary benefits (15).

Bajwa *et al*. compared nitroglycerin, esmolol, and dexmedetomidine as hypotensive anesthetic agents in patients undergoing functional endoscopic sinus surgery, finding that dexmedetomidine and esmolol offered superior hemodynamic stability and surgical field visibility to nitroglycerin (16).

While some studies have reported that hypotensive anesthesia agents have different effects, others have reported no differences. For example, Goswami *et al*. compared efficacy of dexmedetomidine and clonidine to produce hypotensive anesthesia in patients undergoing orthognatic surgery. The results of their study showed no significant difference between the 2 groups for any parameter such as duration of surgery, quality of surgical field, amount of blood loss, need for blood transfusion, total and rescue analgesia used, and associated adverse effects (17). Degoute *et al*. compared the effects of remifentanil, sodium nitroprusside, and esmolol for controlled hypotensive anesthesia. They found that demographic characteristics, hypotension duration, anesthesia duration, and basic hemodynamic variables did not differ significantly among the groups (18).

In our study, while total blood loss and surgical field visibility did not differ among groups, esmolol provided better hemodynamic stability than nitroglycerin. In addition, patients given remifentanil returned more rapidly from a hypotensive to a normotensive state during recovery. A limited number of studies have investigated the effects of hypotensive agents on postoperative pain. A network meta-analysis by Kim *et al*. investigating the effects of hypotensive agents on bleeding and postoperative recovery in nasal surgery reported that postoperative pain outcomes were better with perioperative dexmedetomidine than placebo and other agents (clonidine, dexmedetomidine, beta-blockers, nitroglycerine, and opioids) (19). Another systematic review by Watts *et al*. reported that the perioperative administration of esmolol as a hypotensive agent reduced pain intensity, opioid requirements, rescue analgesic needs, and postoperative nausea and vomiting during the early postoperative period (0-6 hours) (20). In our study, while postoperative pain levels did not differ significantly between groups, they tended to decrease within the first 24 hours, and all patients reported a significant reduction in pain intensity after 12 hours.

In conclusion, remifentanil, esmolol, and nitroglycerin can be used safely for hypotensive anesthesia in orthognathic surgery. Remifentanil and esmolol were more effective than nitroglycerin in perioperative blood pressure control and hemodynamic stability. The anesthesiologist should select the appropriate agent based on the patient’s overall health status.

## Figures and Tables

**Table 1:** Operation related results.

Group	Group 1		Group 2		Group 3		Test Is.	*p*
	Mean ± SD	Median (Min - Max)	Mean ± SD	Median (Min - Max)	Mean ± SD	Median (Min - Max)		
Bleeding	448.00 ±	400.00	492.50 ±	450.00	371.50 ±	400.00	2.076^1^	0.354
Amount (ml)	263.63	(100.00 -1200.00)	253.17	(150.00 -1250.00)	161.52	(120.00 -740.00)	-	-
Intraoperative	1912.50	1900.00	2110.00	2100.00	1867.50	1950.00	1.714^2^	0.189
fluid replacement (ml)	± 369.52	(1200.00 -2500.00)	± 570.92	(1150.00 -3500.00)	± 346.51	(1200.00 -2500.00)	-	-
Hgb (g/dL)	14.40 ± 1.65	14.30 (10.40 -16.80)	14.60 ± 2.13	14.55 (11.60 -17.80)	13.90 ± 1.50	13.70 (11.70 -16.80)	0.879^2^	0.423
INR	0.95 ± 0.07	0.95 (0.82- 1.06)	0.96 ± 0.07	0.94 (0.86- 1.14)	0.99 ± 0.06	0.98 (0.91- 1.13)	3.678^1^	0.159
Plt (10^3/µL)	279.50 ± 63.68	275.50 (157.00 -434.00)	273.65 ± 60.48	274.00 (157.00 -404.00)	294.35 ± 54.10	285.00 (191.00 -413.00)	0.642^2^	0.530
Anesthesia time (min)	242.25 ± 48.14	240.00 (150.00 -340.00)	250.75 ± 52.57	245.00 (145.00 -350.00)	237.50 ± 50.22	220.00 (160.00 -350.00)	1.774^1^	0.412
Surgery time (min)	219.00 ± 46.61	220.00 (120.00 -300.00)	223.25 ± 50.61	220.00 (120.00 -320.00)	213.75 ± 45.94	200.00 (135.00 -320.00)	0.198^2^	0.820

^1 ^Kruskal-Wallis H test, ^2 ^One-way analysis of variance.

**Table 2:** Comparison of heart rate values by groups.

Groups	Group 1		Group 2		Group 3		Test Is.	*p*
	Mean ± SD	Median (Min - Max)	Mean ± SD	Median (Min - Max)	Mean ± SD	Median (Min - Max)		
T0	94.40 ± 19.96	94.00 (52.00- 138.00)	81.75 ± 13.58	82.00 (55.00- 106.00)	84.75 ± 16.57	86.00 (60.00- 118.00)	3.057 ^1^	0.055
T1	83.75 ± 10.72	84.00 (60.00- 99.00)	80.55 ± 21.20	85.50 (12.00- 104.00)	85.95 ± 13.25	87.50 (62.00- 109.00)	0.371 ^2^	0.831
T2	84.55 ± 12.22	84.50 (65.00- 106.00)	84.30 ± 11.01	82.50 (63.00- 112.00)	83.05 ± 15.75	82.00 (58.00- 115.00)	0.075 ^1^	0.928
T3	78.30 ± 12.95	79.00 (57.00- 104.00)	76.70 ± 10.80	76.00 (56.00- 95.00)	84.60 ± 15.91	82.50 (63.00- 112.00)	1.947 ^1^	0.152
T4	80.45 ± 10.25 b	80.50 (58.00- 97.00)	83.35 ± 11.55 b	82.50 (65.00- 106.00)	94.70 ± 13.73 a	93.00 (73.00- 119.00)	7.969 ^1^	0.001
T5	82.70 ± 10.95	81.00 (59.00- 101.00) ab	78.15 ± 11.93	77.00 (65.00- 114.00) a	94.20 ± 14.70	94.50 (74.00- 120.00) b	13.0362	0.003
T6	84.40 ± 13.04 b	86.00 (63.00- 104.00)	80.05 ± 11.79 b	77.00 (62.00- 104.00)	95.05 ± 13.52 a	97.00 (70.00- 116.00)	7.267 ^1^	0.002
T7	79.70 ± 9.96 a	80.50 (64.00- 98.00)	75.80 ± 8.41 a	75.50 (60.00- 93.00)	92.45 ± 14.57 b	92.00 (67.00- 118.00)	9.630 ^1^	<0.001
T8	79.50 ± 12.64 b	83.50 (57.00- 104.00)	78.85 ± 10.13 b	80.00 (61.00- 97.00)	92.00 ± 14.24 a	90.50 (67.00- 117.00)	7.084 ^1^	0.002
T9	81.80 ± 11.20 b	81.50 (66.00- 100.00)	75.20 ± 10.22 b	74.50 (56.00- 97.00)	93.15 ± 10.92 a	94.00 (72.00- 110.00)	14.163^1^	<0.001
T10	79.65 ± 11.55 b	77.00 (60.00- 104.00)	77.35 ± 10.58 b	77.50 (57.00- 98.00)	93.00 ± 10.07 a	94.50 (73.00- 113.00)	12.354^1^	<0.001
T11	79.25 ± 11.67 b	80.00 (62.00- 101.00)	78.30 ± 12.32 b	77.00 (56.00- 105.00)	92.20 ± 11.78 a	93.50 (70.00- 112.00)	8.475 ^1^	0.001
T12	89.40 ± 14.41	89.50 (61.00- 121.00)	88.35 ± 10.17	87.50 (74.00- 104.00)	93.70 ± 14.90	95.00 (67.00	0.904 ^1^	0.410
T13	99.20 ± 15.28	101.50 (65.00 -120.00)	96.60 ± 14.38	98.00 (76.00- 125.00)	97.05 ± 14.87	97.00 (71.00- 124.00)	0.175 ^1^	0.840
T14	101.05 ± 17.81	100.00 (74.00 -140.00)	96.55 ± 12.69	95.50 (76.00- 124.00)	99.70 ± 12.41	103.00 (70.00 -120.00)	0.506 ^1^	0.606
T15	97.00 ± 14.32	99.00 (72.00- 123.00)	93.50 ± 13.95	93.50 (66.00- 124.00)	95.20 ± 15.13	98.00 (60.00- 120.00)	0.292 ^1^	0.748
T16	93.90 ± 16.85	94.00 (58.00- 120.00)	89.60 ± 12.33	89.00 (70.00- 110.00)	92.60 ± 13.44	95.00 (64.00- 112.00)	0.473 ^1^	0.626
T17	91.30 ± 16.84	90.00 (58.00- 126.00)	90.35 ± 15.91	90.00 (65.00- 122.00)	88.55 ± 11.80	91.00 (60.00- 106.00)	0.173 ^1^	0.841
T18	91.40 ± 13.61	90.00 (60.00- 122.00)	90.35 ± 11.37	89.00 (70.00- 116.00)	90.90 ± 10.78	91.00 (65.00- 105.00)	0.038 ^1^	0.962
T19	91.30 ± 11.62	91.00 (72.00- 115.00)	84.80 ± 13.50	82.00 (66.00- 112.00)	85.75 ± 9.35	89.00 (66.00- 100.00)	1.828 ^1^	0.170
T20	87.95 ± 10.68	87.50 (70.00- 110.00)	88.30 ± 11.59	85.50 (70.00- 114.00)	90.55 ± 11.59	92.00 (70.00- 110.00)	0.312 ^1^	0.733

^1 ^One-way analysis of variance, ^2 ^Kruskall Wallis H test, a-b: There is no difference between groups with the same letter.

**Table 3:** Comparison of mean arterial blood pressure values by groups.

Group	Group 1		Group 2		Group 3		Test Is.	*p*
	Mean ± SD	Median (Min - Max)	Mean ± SD	Median (Min - Max)	Mean ± SD	Median (Min - Max)		
T0	91.15 ± 11.79	90.00 (70.00 - 118.00)	91.85 ± 15.99	89.00 (66.00 - 126.00)	87.00 ± 14.85	85.50 (63.00 - 129.00)	2.199 1	0.33 3
T1	82.20 ± 14.29	81.00 (63.00 - 120.00)	87.20 ± 13.05	85.50 (63.00 - 112.00)	89.95 ± 21.59	90.00 (60.00 - 139.00)	1.102 2	0.33 9
T2	78.60 ± 10.95	81.50 (43.00 - 92.00) a	81.70 ± 11.85	83.00 (58.00 - 108.00) a	70.65 ± 10.04	69.00 (55.00 - 88.00) b	9.993 1	0.00 7
T3	77.60 ± 11.71	76.00 (62.00 - 98.00)	80.00 ± 12.93	77.00 (64.00 - 106.00)	72.40 ± 11.16	69.00 (60.00 - 108.00)	4.734 1	0.09 4
T4	81.10 ± 9.26 ab	81.00 (65.00 - 96.00)	90.65 ± 17.43 a	83.00 (63.00 - 123.00)	77.65 ± 12.01 b	77.50 (62.00 - 108.00)	3.732 2	0.03 4
T5	81.95 ± 9.65	82.50 (66.00 - 100.00)	84.40 ± 17.37	84.00 (58.00 - 118.00)	82.75 ± 12.73	81.50 (63.00 - 114.00)	0.151 2	0.86 0
T6	83.85 ± 15.19	79.00 (65.00 - 113.00)	87.55 ± 11.76	87.50 (67.00 - 106.00)	84.00 ± 11.99	82.50 (62.00 - 116.00)	0.513 2	0.60 1
T7	72.45 ± 11.20	68.50 (62.00 - 97.00)	74.15 ± 9.26	74.50 (60.00 - 95.00)	72.50 ± 8.34	71.50 (59.00 - 90.00)	0.977 1	0.61 4
T8	72.70 ± 11.59	68.50 (60.00 - 92.00)	77.90 ± 13.75	74.50 (56.00 - 106.00)	71.45 ± 9.64	68.00 (60.00 - 90.00)	2.642 1	0.26 7
T9	75.40 ± 10.11	74.00 (59.00 - 92.00)	74.75 ± 9.28	74.50 (59.00 - 94.00)	70.60 ± 8.48	70.00 (59.00 - 89.00)	1.563 2	0.21 8
T10	76.40 ± 13.36	73.00 (57.00 - 111.00)	74.95 ± 13.95	73.50 (53.00 - 111.00)	72.70 ± 7.62	75.00 (61.00 - 88.00)	0.484 2	0.61 9
T11	73.80 ± 10.69	69.00 (60.00 - 97.00)	71.25 ± 9.77	68.00 (59.00 - 101.00)	71.90 ± 10.44	69.50 (60.00 - 97.00)	0.707 1	0.70 2
T12	85.30 ± 14.64 a	85.00 (56.00 - 105.00)	74.20 ± 12.83 b	71.00 (57.00 - 106.00)	79.05 ± 10.31 ab	77.50 (65.00 - 103.00)	3.828 2	0.02 8
T13	92.50 ± 15.08 a	93.00 (64.00 - 118.00)	79.25 ± 12.39 b	80.00 (52.00 - 101.00)	79.90 ± 9.11 b	80.50 (64.00 - 100.00)	7.216 2	0.00 2
T14	85.70 ± 14.26	85.50 (73.00 - 126.00)	78.55 ± 8.13	76.00 (67.00 - 95.00)	82.05 ± 10.57	79.50 (72.00 - 103.00)	2.399 1	0.30 1
T15	86.60 ± 11.63	84.50 (73.00 - 113.00)	80.55 ± 10.20	80.50 (70.00 - 110.00)	83.05 ± 10.30	83.00 (70.00 - 103.00)	3.907 1	0.14 2
T16	86.10 ± 12.21	83.00 (73.00 - 113.00)	82.35 ± 8.38	83.00 (70.00 - 100.00)	84.25 ± 9.69	83.00 (73.00 - 103.00)	0.498 1	0.78 0
T17	84.75 ± 11.37	83.00 (70.00 - 120.00)	79.25 ± 6.52	83.00 (70.00 - 93.00)	82.50 ± 7.75	81.50 (73.00 - 96.00)	2.681 1	0.26 2
T18	80.75 ± 7.17	83.00 (70.00 - 93.00)	78.75 ± 5.40	80.00 (70.00 - 90.00)	81.60 ± 7.47	83.00 (70.00 - 93.00)	1.588 1	0.45 2
T19	77.90 ± 6.46	74.50 (70.00 - 93.00)	78.60 ± 7.86	76.00 (70.00 - 93.00)	75.95 ± 7.42	73.00 (70.00 - 93.00)	2.673 1	0.26 3
T20	80.20 ± 8.19	80.00 (70.00 - 96.00)	76.35 ± 6.92	73.00 (70.00 - 93.00)	78.65 ± 7.64	73.50 (70.00 - 93.00)	2.880 1	0.23 7

^1 ^Kruskall Wallis H test, ^2 ^One-way analysis of variance, a-b: There is no difference between the groups with the same letter.
